# Similar Morphologies but Different Origins: Hybrid Status of Two More Semi-creeping Taxa of *Melastoma*

**DOI:** 10.3389/fpls.2017.00673

**Published:** 2017-04-26

**Authors:** Peishan Zou, Wei Lun Ng, Wei Wu, Seping Dai, Zulin Ning, Shuqiong Wang, Ying Liu, Qiang Fan, Renchao Zhou

**Affiliations:** ^1^State Key Laboratory of Biocontrol and Guangdong Provincial Key Laboratory of Plant Resources, School of Life Sciences, Sun Yat-sen UniversityGuangzhou, China; ^2^Guangzhou Institute of Forestry and Landscape ArchitectureGuangzhou, China; ^3^Guangdong Provincial Key Laboratory of Applied Botany, South China Botanical Garden, Chinese Academy of SciencesGuangzhou, China

**Keywords:** natural hybridization, *Melastoma*, molecular analysis, introgression, nuclear genes

## Abstract

Inferring the origins of hybrid taxa based on morphology alone is difficult because morphologically similar hybrids can arise from hybridization between different populations of the same parental species or be produced by hybridization of different parental species. In this study, we investigated the origins of two semi-creeping taxa in *Melastoma*, which are morphologically similar to a natural hybrid, *M. intermedium*, by sequencing a chloroplast intergenic spacer, nuclear ribosomal internal transcribed spacer and two low-copy nuclear genes (*tpi* and *cam*) in these taxa and their putative parental species. Our sequence analysis provides compelling evidence for the hybrid status of the two semi-creeping taxa: one originating from hybridization between *M. dodecandrum* and *M. malabathricum*, and the other between *M. dodecandrum* and *M. normale*. The origins of these hybrids are therefore clearly different from *M. intermedium*, and morphological similarity for the three hybrids is most likely due to their origins from hybridization between the same creeping species *M. dodecandrum* and a different erect species in each of the three cases. We also observed low rate of introgression from *M. normale* to *M. dodecandrum*, and genetic exchange between them may transfer adaptive traits to *M. dodecandrum*. Rare occurrence of these two hybrids may be due to small range overlaps between parental species in one case, and different flowering periods between parental species in the other.

## Introduction

Natural hybridization is pervasive in flowering plants and it can generate evolutionary novelty through the formation of hybrid lineages or interspecific introgression (Arnold, 1997; Rieseberg et al., 1998; Goulet et al., 2017). When one species hybridizes with another, hybrids can establish locally and then may disperse to other locations where one or both parental species do not occur (e.g., Fitzpatrick et al., 2010). If parental species overlap in geographic distribution in multiple locations, hybrids may arise independently from hybridization between local populations of parental species (e.g., Zhang et al., 2013). Under this circumstance, hybrids from different locations may show subtle or obvious morphological differences. This can be caused by morphological divergence in one or two parental species from different locations, or environmental differences between different locations. On the other hand, when one species hybridizes with two or more species, hybrids produced from different combinations of parental species may also show subtle or obvious morphological differences. Thus, different hybrid taxa with slight morphological dissimilarity may have arose from hybridization of the same parental species in different locations or hybridizations involving different combinations of parental species. Therefore, inferring the origins of such hybrid taxa based on morphology alone is difficult. In such cases, molecular means have been proven successful in determining the origins of various hybrid taxa (Zhang et al., 2013; Harrison and Larson, 2014).

*Melastoma*, a shrub genus distributed in tropical Asia and Oceania, has undergone rapid adaptive radiation, with more than 20 species diversified during the last one million years (Meyer, 2001; Renner and Meyer, 2001). Wong (2016), however, estimated that there are 80–90 species in this genus. Because there is substantial overlap in geographic distribution and flowering time between some species of this genus, interspecific hybridization is assumed to be relatively common (Dai et al., 2012; Liu et al., 2014). However, only two cases of natural hybridization have been confirmed using molecular means so far (Dai et al., 2012; Liu et al., 2014), and the role of natural hybridization in the evolution of this genus remains elusive.

*Melastoma dodecandrum* is the only creeping species in the genus, while the other species are all erect shrubs. Previously, we identified *M. intermedium*, a semi-creeping shrub, as a natural hybrid between *M. dodecandrum* and *M. candidum* (Dai et al., 2012). During our field survey of *Melastoma* in Guangdong in the past few years, we found two more semi-creeping taxa of *Melastoma*, one in Ruyuan and the other in Xinyi. Both taxa are of very rare occurrence. These two taxa have the similar stem type (i.e., semi-creeping) as *M. intermedium*, but there are subtle morphological differences between these three semi-creeping taxa: the taxon found in Ruyuan and *M. intermedium* have very short appressed scales on the young twigs, while the taxon found in Xinyi has spreading bristles on the young twigs. The taxon found in Xinyi and *M. intermedium* has oblong leaves and strigose upper surfaces, while the taxon found in Ruyuan possesses lanceolate leaves and subglabrous upper surfaces (Supplementary Table S1). At the sampling site of Ruyuan, only *M. dodecandrum* and *M. malabathricun* coexist, while at the sampling site of Xinyi, only *M. dodecandrum* and *M. normale* co-occur. There was no *M. candidum*, one of the parental species of *M. intermedium*, at both sites.

The semi-creeping nature of the two newly found taxa results likely from separate hybridization events between the creeping *M. dodecandrum* and the erect *M. malabathricum* in Ruyuan, and *M. normale* in Xinyi. In this scenario, morphological differences between the three semi-creeping taxa may stem mainly from morphological differences among one of their parental species, *M. candidum*, *M. malabathricun*, and *M. normale*, since they share the other parental species, *M. dodecandrum*. However, there is also a possibility that these two taxa are in fact *M. intermedium*, germinated from seeds brought by birds from other locations where *M. dodecandrum* and *M. candidum* co-occur and hybridize. Birds have been known to be seed dispersers for *Melastoma* species (Whittaker and Jones, 1994; Westcott et al., 2008). Morphological differences in this scenario may be caused by environmental differences.

Figuring out the status of a taxon in question is crucial for understanding its taxonomy and evolution. For this study, we collected samples from populations of the possible parental species *M. dodecandrum*, *M. normale*, *M. malabathricum*, and *M. candidum*, as well as the two unidentified taxa (designated as DM and DN hereafter, from Ruyuan and Xinyi, respectively). We sequenced one chloroplast intergenic spacer, nuclear ribosomal internal transcribed spacer (nrITS) and two low-copy nuclear genes (*tpi* and *cam*) to investigate the taxonomic status of the two newly found semi-creeping *Melastoma* taxa. Because introgression is a common consequence of hybridization and can contribute to transfer of adaptation (Arnold and Martin, 2009; Whitney et al., 2010), we also aimed to examine if there is any evidence of introgression between the putative parental species.

## Materials and Methods

### Plant Materials

**FIGURE 1:**
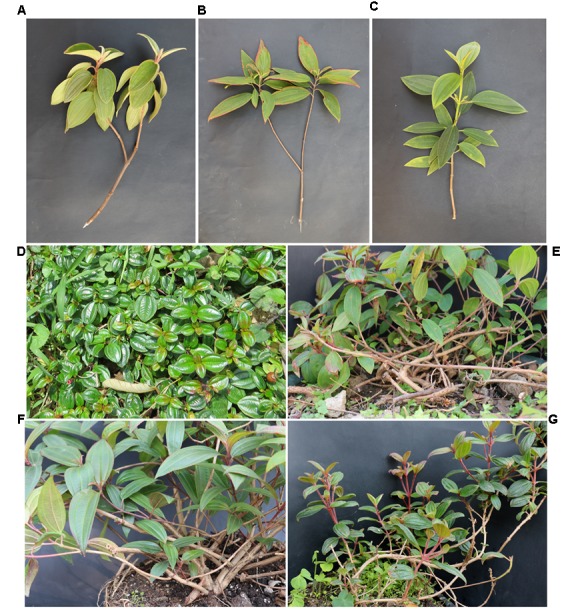
**Morphological comparison between three putative hybrids and their putative parental species in *Melastoma*. (A)** *M. candidum*, **(B)** *M. malabathricum*, **(C)** *M. normale*, **(D)** *M. dodecandrum*, **(E)** *M. intermedium*, **(F)** Putative hybrid between *M. dodecandrum* and *M. malabathricum*, and **(G)** Putative hybrid between *M. dodecandrum* and *M. normale.*

We sampled one population each of *M. dodecandrum*, *M. malabathricum*, and the unidentified taxon (DM) in Tianjingshan, Ruyuan, Guangdong, China, and one population each of *M. dodecandrum*, *M. normale*, and the other unidentified taxon (DN) in Dawuling, Xinyi, Guangdong. All three erect species occur in open habitats, while *M. dodecandrum* prefers slightly shady habitats. DM occurs along several trailsides near Tianjinshan, Ruyuan, with the elevations of 500–700 m. DN was found along the roadside in the Dawuling Nature Reserve, Xinyi, at an altitude of about 900–1000 m. It seems that both locations have experienced human disturbance due to road or trail constructions. Only four to five samples of the unidentified taxa were collected due to their scarcity in both locations. The sampling details are shown in **Table 1**. To test whether the two unidentified taxa are *M. intermedium* or not, we also collected samples of *M. candidum* from Ziyun Park, Longhai, Fujian, China. Morphological comparisons of these taxa are shown in **Figure 1** and Supplementary Figures S1–S3 and Table S1. Leaves were sampled and dried in plastic bags with silica gel for subsequent DNA extraction.

**Table 1:** Sampling details of six taxa of *Melastoma* used in this study.

Taxon	Locality	Sample size
*M. dodecandrum* (D_R_)	Tianjingshan, Ruyuan, Guangdong	20
*M. dodecandrum* (D_X_)	Dawuling, Xinyi, Guangdong	20
*M. malabathricum* (M)	Tianjinshan, Ruyuan, Guangdong	18
*M. normale* (N)	Dawuling, Xinyi, Guangdong	20
*M. candidum* (C)	Ziyun Park, Longhai, Fujian	20
Putative hybrid between D and M (DM)	Tianjinshan, Ruyuan, Guangdong	5
Putative hybrid between D and N (DN)	Dawuling, Xinyi, Guangdong	4

### DNA Extraction, PCR, and Sequencing

We extracted genomic DNA from dried leaf tissue using the CTAB method (Doyle and Doyle, 1987). We amplified the chloroplast *trn*L-*trn*F intergenic spacer and nrITS regions using the universal primers trn*-*c and trn*-*f (Taberlet et al., 1991), and ITS4 and ITS5 (White et al., 1990), respectively. Two low-copy nuclear genes (*tpi* and *cam*), which encode triose phosphate isomerase and calmodulin, respectively, were PCR-amplified and sequenced using the primer sequences from Dai et al. (2012) and Chao et al. (2014), respectively. We purified the PCR products using the Pearl Gel Extraction Kit (Pearl Bio-tech, Guangzhou, China) and then directly sequenced them on an ABI 3730 DNA automated sequencer with the BigDye chemistry (Applied Biosystems, Foster City, CA, USA). For sequences with more than one polymorphic site and insertion/deletion polymorphisms, cloning-sequencing was conducted to phase the haplotypes. We conducted ligation reactions with a pMD18-T&A cloning kit (Takara, Dalian, China) and selected eight positive colonies for each individual for sequencing. We deposited all the sequences in GenBank with accession numbers KY798014–KY798110.

### Sequence Analyses

We edited the sequences using SeqMan (DNASTAR Inc., Madison, WI, USA) and aligned them using Clustal X (Thompson et al., 1997). The haplotypes of the nuclear loci that had no, or one, polymorphic site, were inferred using Phase (Librado and Rozas, 2009). We used the median-joining method (Bandelt et al., 1999) implemented in Network 4.6^1^ to resolve the relationships among the haplotypes of each locus.

## Results

### Sequence Analysis of Chloroplast *trn*L-*trn*F

**FIGURE 2:**
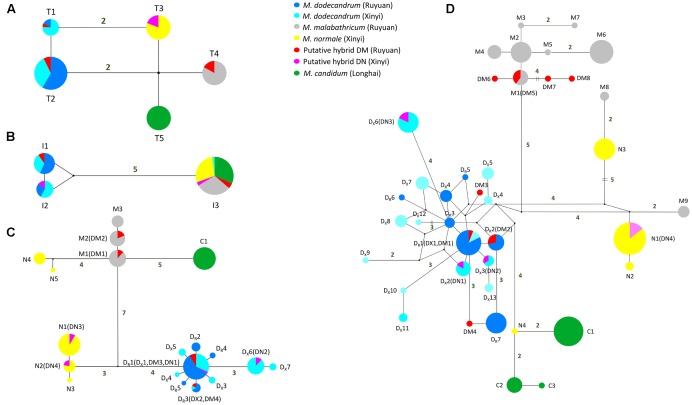
**Median-joining networks of (A)** chloroplast *trn*L*-trn*F region, **(B)** nrITS region, **(C)** *tpi* gene, and **(D)** *cam* gene. For the two low-copy nuclear genes, haplotypes of each taxon are denoted using the first letter of its species name (except *M. dodecandrum* for which ‘D_R_’ and ‘D_X_’ are used to denote the Ruyuan and Xinyi populations, respectively) followed by a number (**Table 1**). Small black circles represent hypothetical haplotypes. Mutational steps are shown by the number near the connecting lines, and the number is omitted for those with only one mutational step.

The aligned length of chloroplast *trn*L*-trn*F in all samples was 825 bp. We detected four nucleotide substitutions and one insertion/deletion (indel) in these samples, which generated five haplotypes (Supplementary Table S2 and **Figure 2**). In each of the two populations (Ruyuan and Xinyi) of *M. dodecandrum*, we observed the same two haplotypes (T1 and T2), with one mutational step between them. For the three other species, *M. malabathricum*, *M. normale*, and *M. candidum*, only one unique haplotype for each species was detected. *Melastoma dodecandrum*, *M. malabathricum*, *M. normale*, and *M. candidum* can be distinguished from each other because there were no shared haplotypes between them. There were at least two mutational steps between haplotypes of any two of the putative parental species. As for DM and DN, no private haplotypes were found. Three of the five individuals of DM shared the haplotype with *M. malabathricum*, and the remaining two individuals shared the haplotypes of *M. dodecandrum*. All four individuals of DN had the same haplotype as *M. normale* (Supplementary Table S2). Neither DM nor DN shared haplotypes with *M. candidum*.

### Sequence Analysis of nrITS Region

The length of nrITS regions in the six taxa of *Melastoma* was 691 bp after sequence alignment. There were five nucleotide substitutions and one 1-bp indel across these samples, which led to three haplotypes (**Figure 2**). No intraspecific variation was found for *M. malabathricum*, *M. normale*, and *M. candidum*, and they shared the same haplotype I3. Except one individual of *M. dodecandrum* from Xinyi population, which had the I3 haplotype (it should be introgressed from *M. normale* and we will discuss it later), both populations (Ruyuan and Xinyi) of *M. dodecandrum* had two haplotypes, I1 and I2. Only one mutational step existed between I1 and I2. There were six mutational steps between haplotype I3 and the haplotypes of *M. dodecandrum* (I1 and I2). All the sampled individuals of DM and DN were heterozygous at this region, with all four individuals of DM possessing I1 and I2, and all five individuals of DN possessing I1 and I3 (Supplementary Table S3 and **Figure 2**).

### Sequence Analysis of Two Low-Copy Nuclear Genes

***tpi*** *–* The partial *tpi* gene was 703 bp in length after sequence alignment. A total of 32 variable sites at this gene were detected. There were 11 differentially fixed sites between *M. malabathricum* and *M. dodecandrum* from Ruyuan, and on each of these sites, DM showed chromatogram signal additivity (Supplementary Table S4). Likewise, there were four sites differentially fixed in *M. normale* and *M. dodecandrum* from Xinyi, and on each of these sites, DN exhibited chromatogram signal additivity (Supplementary Table S4). In the haplotype analysis, no shared haplotypes were observed among the four species. Except for *M. normale*, haplotypes of each species formed a cluster (**Figure 2**). Haplotypes of *M. normale* formed two highly divergent groups. All the haplotypes detected in DM were shared with *M. dodecandrum* from Ruyuan and *M. malabathricum* (**Figure 2** and Supplementary Table S4). Likewise, all the haplotypes found in DN were shared with either *M. dodecandrum* from Xinyi or *M. normale*.

***cam*** *–* The aligned sequence length of the partial *cam* gene was 741 bp. There was only one differentially fixed site (the 70th site: T for *M. dodecandrum* and G for *M. normale*) between *M. normale* and *M. dodecandrum* from Xinyi, and on this site, DN showed chromatogram signal additivity. However, no differentially fixed sites were observed between *M. malabathricum* and *M. dodecandrum* from Ruyuan. In the haplotype analysis, *M. dodecandrum* and *M. malabathricum* showed more haplotypes than *M. normale* and *M. candidum*. Although *M. malabathricum*, *M. normale*, and *M. candidum* did not form their own distinct clusters, no shared haplotypes were observed between any two species. Two haplotypes of *M. malabathricum* (M8 and M9) were more closely related to those of *M. normale*, and one haplotype of *M. normale* (N4) was more closely related to those of *M. candidum.* All the haplotypes identified in DN were shared with *M. dodecandrum* from Xinyi and *M. normale*, while three of eight haplotypes detected in DM were shared with *M. dodecandrum* from Ruyuan and *M. malabathricum*. Two and three private haplotypes were closely related to those of *M. dodecandrum* and *M. malabathricum*, respectively.

## Discussion

### Molecular Evidence for Hybrid Status of the Two Semi-creeping Taxa of *Melastoma*

The two semi-creeping taxa of *Melastoma* (DM and DN) sampled in this study resemble another previously identified hybrid *M. intermedium* in morphology, but we were not certain if they had the same origin or not. In this study, we obtained sequence data from chloroplast DNA and nuclear DNA to clarify their taxonomic status. At the chloroplast locus, the individuals of DM had the same haplotype as either *M. malabathricum* or *M. dodecandrum*, and those of DN had identical haplotype with *M. normale* only. At all three nuclear loci, each individual of DN had two haplotypes, each matching those of *M. dodecandrum* and *M. normale* (**Table 2**). At two of the three nuclear loci, each individual of DM had two haplotypes, each matching those of *M. dodecandrum* and *M. malabathricum*. Although DM had several private haplotypes at the *cam* gene, these private haplotypes were clustered together with those of *M. dodecandrum* or *M. malabathricum*. Considering that *M. dodecandrum* and *M. malabathricum* had relatively high levels of polymorphism at this gene, unsampled polymorphisms in the parental species are likely to have produced this pattern. Of course, we could not exclude the possibility of new mutations occurring in DM. Therefore, molecular data in our study provided compelling evidence for the hybrid status of the two semi-creeping taxa of *Melastoma*. The two hybrids originated from hybridization between *M. dodecandrum* and *M. malabathricum* (DM), and between *M. dodecandrum* and *M. normale* (DN), different from the origins of *M. intermedium*. Morphological similarity for the three hybrids is due likely to their origins from hybridization between the same creeping species, *M. dodecandrum*, and a different erect species, in each of the three cases. Because all the individuals of the two hybrids had two divergent haplotypes corresponding to their parental species at each of the three nuclear loci, they were all probably F_1_ hybrids. It is also possible that they are later-generation hybrids but more nuclear loci should be analyzed to be certain.

**Table 2:** Genotypes of the two hybrids of *Melastoma* at one chloroplast intergenic spacer (*trn*L-*trn*F), nrITS region, and two nuclear genes (*tpi* and *cam*).

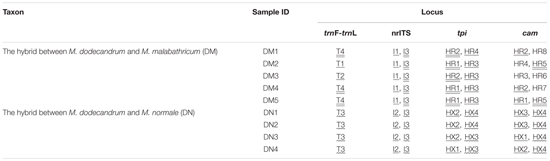

*Genotypes with single, double, or wavy underlines have identical sequences with those of M. dodecandrum, M. malabathricum, and M. normale, respectively. Genotypes without underline are unique to hybrids.*

Previous studies have also identified natural hybridization in other combinations of *Melastoma* species (Dai et al., 2012; Liu et al., 2014). Along with this study, we can see that natural hybridization between species of *Melastoma* is relatively common. Substantial overlap in geographic distribution and flowering time, as well as shared pollinators, may facilitate the occurrence of natural hybridization in *Melastoma* (Dai et al., 2012; Liu et al., 2014). As for the focus species in our study, *M. dodecandrum* is distributed in southern China and northern Vietnam, while *M. normale* ranges from northern Southeast Asia to southern China. *M. malabathricum* is the most widespread species throughout the distribution range of *Melastoma*, but in China, it is confined to the northern edge of southern China. Despite the overlap in geographic distribution of each pair of parental species, we failed to find the two hybrids in many locations where their parental species overlap. The rare occurrence of DM may be due to the relatively small range overlap of its parental species. In the case of DN, it may be caused by the difference in flowering period between its parental species. In China, *M. dodecandrum* flowers from May to July, and *M. normale* flowers usually from March to April. But in locations of high elevation like Dawuling in Xinyi, the lower temperature delays the flowering time for *M. normale*, leading to an overlap of flowering time between *M. normale* and *M. dodecandrum.* In fact, a small fraction of individuals of *M. normale* can flower in May (R. Zhou, personal observation), providing temporal opportunity for hybridization to occur. Human disturbance due to road or trail constructions may also facilitate the occurrence of hybridization between them. From our observations at the sampling sites, the two newly-discovered hybrids from Xinyi and Ruyuan, as well as the more common *M. intermedium*, were growing alongside their parental species, suggesting no niche shifts in the hybrids.

### The Role of Natural Hybridization in the Evolution of *Melastoma*

A common outcome of interspecific hybridization is introgression. Although most introgressed loci are neutral, a few loci related to adaptation can be transferred from one species to another and thus lead to rapid adaptation of the recipient species to new environments (Baack and Rieseberg, 2007). *Melastoma dodecandrum* can hybridize with the three other congeneric species, however, the number of individuals for each hybrid is very small, suggesting strong reproductive isolation between *M. dodecandrum* and the three other species. Strong reproductive isolation, however, does not completely hinder interspecific introgression. One individual of *M. dodecandrum* in Xinyi population had the same nrITS haplotype (I3) as the three other species. Haplotype sharing for this individual is due more likely to introgression from the sympatric *M. normale* rather than to ancestral polymorphism in *M. dodecandrum*, as reasoned here. First, the nrITS haplotypes of 39 out of 40 *M. dodecandrum* individuals (I1 and I2) are highly divergent from that of the three other species (I3), with six mutational steps between them. Second, only two haplotypes (I1 and I2) were observed in all 20 individuals of *M. dodecandrum* from Ruyuan. If the extra haplotype (I3) present in the *M. dodecandrum* individual had been due to ancestral polymorphism, we would expect the same haplotype to also be observed in the *M. dodecandrum* population from Ruyuan. We detected introgression to only one individual of *M. dodecandrum* from *M. normale* at only the nrITS locus, suggesting a low rate of introgression to *M. dodecandrum* from *M. normale*. Interestingly, this individual was also homozygous at the nrITS locus, which can be attributed to concerted evolution after hybridization, rapidly homogenizing the nrITS copies within a genome. Concerted evolution of the nrITS region can be observed in as early as the F_2_ generation (Fuertes Aguilar et al., 1999). Because the individual of *M. dodecandrum* does not have alleles of *M. normale* at other loci we studied, it must be a later-generation hybrid. Therefore, it is probable that rapid concerted evolution resulted in the homogenization of the *M. normale* nrITS copy in the individual. Even at a low level of introgression, adaptive alleles and corresponding adaptive traits may be transferred from one species to another, likely contributing to adaptation to various environments (Abbott et al., 2016; e.g., Lamichhaney et al., 2016). The extent of introgression and loci of adaptive introgression in *Melastoma* can be further explored with genomic scanning in the future.

## Author Contributions

RZ, QF, and YL designed the study. RZ, QF, YL, SD, PZ and ZN collected materials, and experiments were performed by PZ and SW. RZ, QF, YL, SD and ZN guided the experiments. PZ analyzed and interpreted the data and wrote the manuscript with guidance of RZ, WLN, and WW. All authors read and approved the final manuscript.

## Conflict of Interest Statement

The authors declare that the research was conducted in the absence of any commercial or financial relationships that could be construed as a potential conflict of interest.

## Footnotes

1 http://www.fluxus-engineering.com
