# Supplementary material for: Similar Morphologies but Different Origins: Hybrid Status of Two More Semi-creeping Taxa of Melastoma
Source: Front Plant Sci. 2017 Apr 26;8:673. doi: 10.3389/fpls.2017.00673 (PMC5405130; doi:10.3389/fpls.2017.00673)
Supplement: Supplementary file 1 [file Presentation_1.PDF]

Supplementary Material

Similar morphologies but different origins: Hybrid status of two more semi-creeping taxa of *Melastoma*

Peishan Zou[1], Wei Lun Ng[1], Wei Wu[1], Seping Dai[2], Zulin Ning[3], Shuqiong Wang[1], Ying Liu[1], Qiang Fan[1], Renchao Zhou[1*]

***Correspondence: Renchao Zhou**

Email: zhrench@mail.sysu.edu.cn

Table S1 Morphological comparisons among seven taxa of *Melastoma* featured in this study.

**Taxon**	**Stem type**	**Leaf shape**	**Indumentum on young twigs**	**Indumentum on leaves**	**Indumentum on hypanthium**	**Fruit**
M. dodecandrum (D)	creeping	ovate to elliptic	strigose or glabrous	sparsely strigose or glabrous	strigose, base of trichomes conically inflated	globose, fleshy indehiscent
M. malabathricum (M)	erect	lanceolate	densely covered with appressed scales	strigose	densely covered with short appressed scales	campanulate, fleshy, transversally dehiscent
M. normale (N)	erect	oblong	densely covered with spreading bristles	densely pilose	densely covered with appressed scales	campanulate, fleshy, transversally dehiscent
M. candidum (C)	erect	ovate	densely covered with appressed scales	densely pilose	densely covered with appressed scales	campanulate, fleshy, transversally dehiscent
M. intermedium (hybrid between D and	semi-creeping	oblong to ovate	sparsely covered with short appressed scales	sparsely strigose	strigose	subglobose, fleshy indehiscent

C)

Putative hybrid between D and M (DM)	semi-creeping	lanceolate	sparsely covered with short appressed scales	subglabrous	strigose	subglobose, fleshy indehiscent
Putative hybrid between D and N (DN)	semi-creeping	oblong to ovate	densely covered with spreading bristles	sparsely strigose	strigose	subglobose, fleshy indehiscent

Table S2 The base composition of variable sites at the chloroplast *trn*L-*trn*F region among putative parental species and the two putative hybrids (DM and DN).

Taxon (sampling location)	Haplotype	The number of individual with this haplotype	Variable sites				
			**220**	**367**	**434**	**607**	**748**
M. dodecandrum (Ruyuan)	T1	2	C	T	A	G	C
	T2	18	C	T	-	G	C
M. malabathricum (Ruyuan)	T4	18	T	C	-	T	C
Putative hybrid DM (Ruyuan)	T1	1	C	T	A	G	C
	T2	1	C	T	-	G	C
	T4	3	T	C	-	T	C
M. dodecandrum (Xinyi)	T1	6	C	T	A	G	C
	T2	14	C	T	-	G	C
M. normale (Xinyi)	T3	20	C	T	A	T	C
Putative hybrid DN (Xinyi)	T3	4	C	C	A	T	C
M. candidum (Longhai)	T5	19	C	C	-	T	T

-: deletion

Table S3 The base composition of variable sites at the nrITS region among putative parental species and the two putative hybrids (DM and DN). Bases with double peaks in the chromatograms are recorded according to the IUPAC ambiguity symbols: M=A+C; R=A+G; W=A+T.

**Taxon Site**	**192**	**193**	**205**	**430**	**436**	**643**
M. dodecandrum (Ruyuan)	C	G	G	A	R	A
M. malabathricum (Ruyuan)	A	A	A	G	-	T
Putative hybrid DM (Ruyuan)	M	R	R	R	G/-	W
M. dodecandrum (Xinyi)	C	G	G	A	R	A
M. normale (Xinyi)	A	A	A	G	-	T
Putative hybrid DN (Xinyi)	M	R	R	R	A/-	W
M. candidum (Longhai)	A	A	A	G	-	T

-: deletion; G/-, A/-: heterozygous at the indel position.

Table S4 The base composition of differentially fixed sites at the *tpi* gene between putative parental species in the two putative hybrids (DM and DN). Bases with double peaks in the chromatograms are recorded according to the IUPAC ambiguity symbols: M=A+C; R=A+G; W=A+T. Differentially fixed sites between parental species are underlined.

**Taxon Site**	**1**	**10**	**204**	**209**	**225**	**258**	**272**	**312**	**340**	**358**	**567**
M. dodecandrum (Ruyuan)	G	C	A	T	C	C	A	A	C	A	T
M. malabathricum (Ruyuan)	C	T	T	A	T	T	G	G	T	T	C
Putative hybrid DM (Ruyuan)	S	Y	W	W	Y	Y	R	R	Y	W	Y
M. dodecandrum (Xinyi)	G	C	A	T	C	C	A	A	C	A	T
M. normale (Xinyi)	C	Y	W	A	Y	Y	R	G	Y	T	Y
Putative hybrid DN (Xinyi)	S	C	A	W	C	C	A	R	C	W	T
M. candidum (Longhai)	C	T	T	A	T	T	G	G	T	T	C

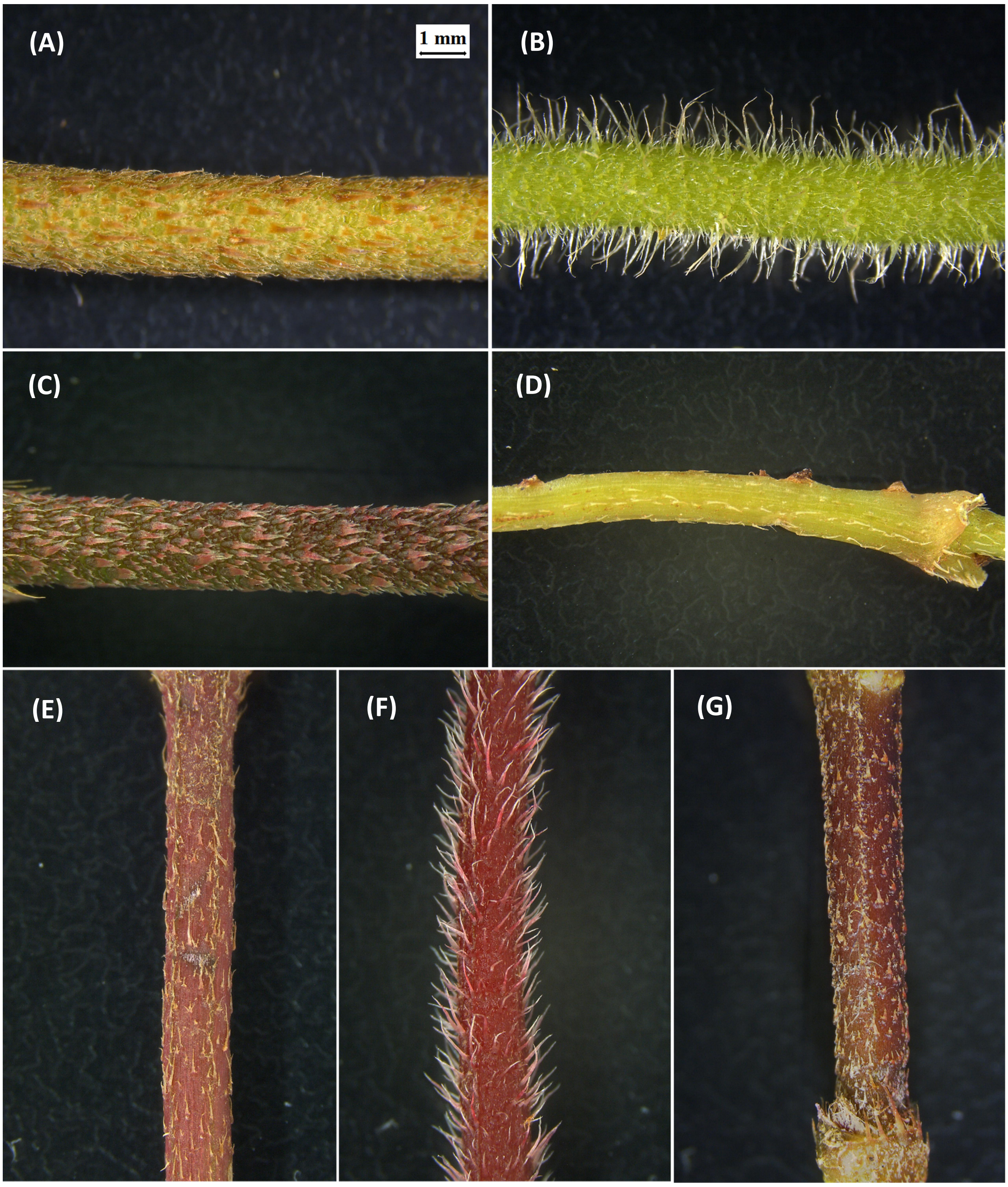

Supplementary Figure 1 The trichomes on the young twigs of (A) *M. candidum*, (B) *M. normale*, (C) *M. malabathricum*, (D) *M. dodecandrum*, (E) *M. intermedium*, (F) putative hybrid between *M. dodecandrum* and *M. normale*, and (G) putative hybrid between *M. dodecandrum* and *M. malabathricum.*

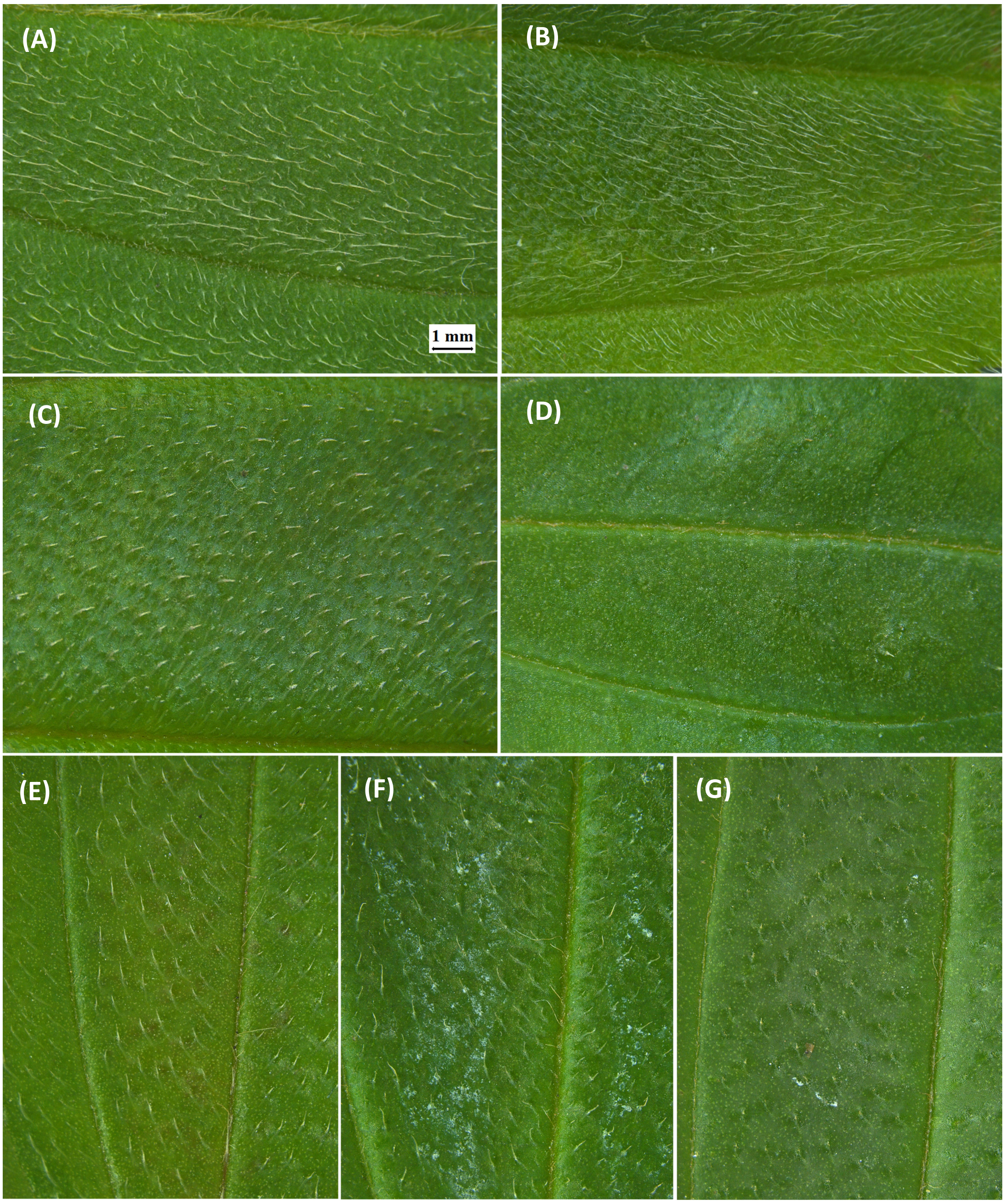

Supplementary Figure 2 The bristles on the leaf surface of (A) *M. candidum*, (B) *M. normale*, (C) *M. malabathricum*, (D) *M. dodecandrum*, (E) *M. intermedium*, (F) putative hybrid between *M. dodecandrum* and *M. normale*, and (G) putative hybrid between *M. dodecandrum* and *M. malabathricum.*

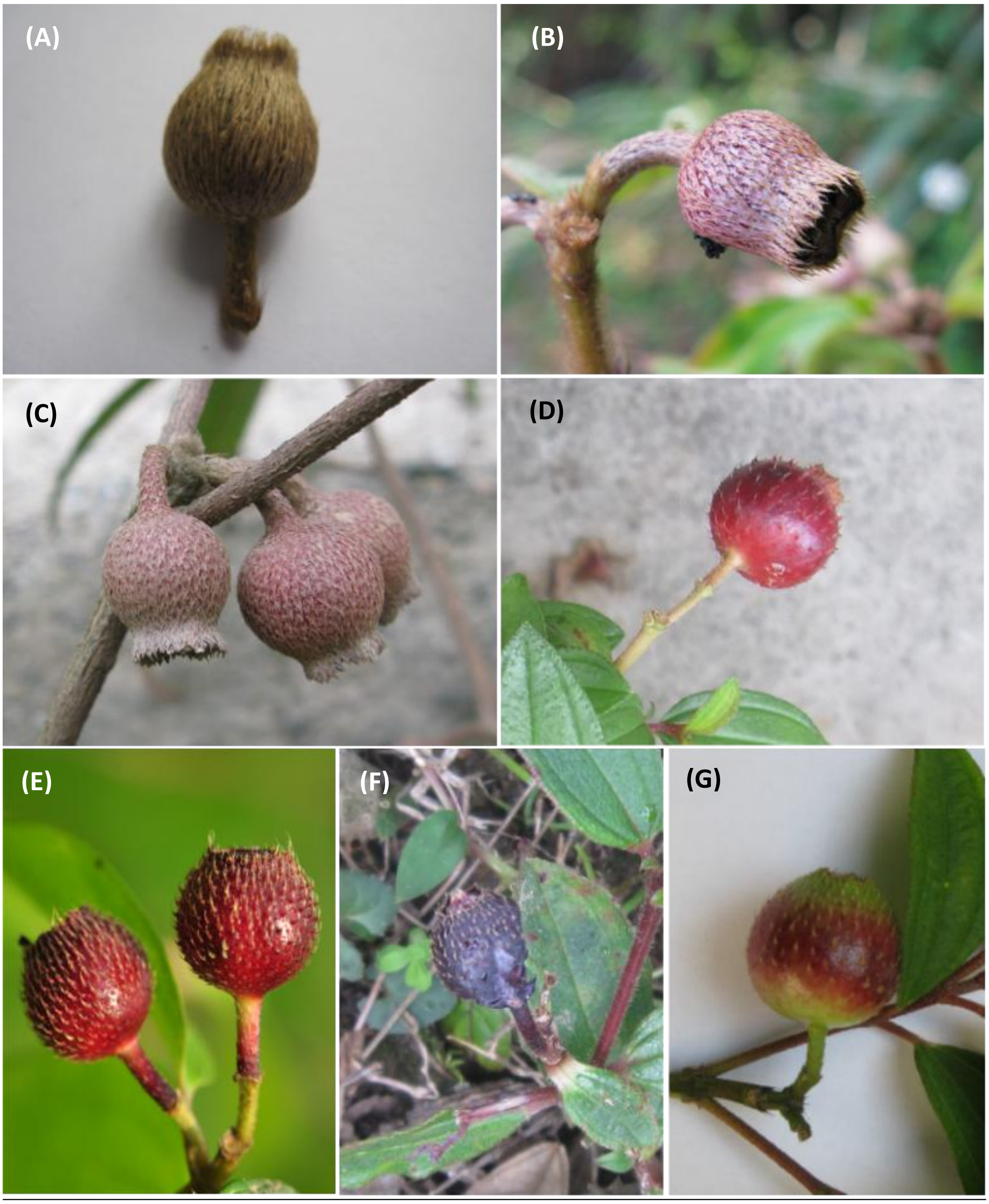

Supplementary Figure 3 The trichomes on the fruits of (A) *M. candidum*, (B) *M. normale*, (C) *M. malabathricum*, (D) *M. dodecandrum*, (E) *M. intermedium*, (F) putative hybrid between *M. dodecandrum* and *M. normale*, and (G) putative hybrid between *M. dodecandrum* and *M. malabathricum.*
